# Neighborhood Cohesion and Mobility Limitations Among Older Americans: The Roles of Depressive Symptoms and Mastery

**DOI:** 10.1093/geroni/igab046.1346

**Published:** 2021-12-17

**Authors:** Fei Wang, Weidi Qin, Jiao Yu

**Affiliations:** 1 Case Western Reserve University, Cleveland Heights, Ohio, United States; 2 University of Michigan; 3 Department of Sociology, College of Arts and Sciences, Case Western Reserve University, Cleveland, Ohio, United States

## Abstract

Neighborhood environment has been playing an important role in late-life health; yet, the social aspect of neighborhood environment and its impact on mobility limitations have been rarely examined. This study examines the relationship between neighborhood social cohesion and mobility limitations and the potential mediators (i.e., depressive symptoms, mastery) of this relationship. A total of 8,317 Americans aged 65 and older were selected from the Health and Retirement Study. Using ordinary least squares (OLS) regressions, this study shows that neighborhood social cohesion is negatively associated with mobility limitations, and this association is mediated by depressive symptoms and mastery respectively. Specifically, neighborhood social cohesion can reduce mobility limitations through mitigating depressive symptoms and increasing mastery. The findings have implications for developing community measures to promote neighborhood social cohesion and applying psychosocial interventions to reduce depressive symptoms and improve mastery among older adults.

